# Supplementary material for: Genome Sequencing of Streptomyces olivaceus SCSIO T05 and Activated Production of Lobophorin CR4 via Metabolic Engineering and Genome Mining
Source: Mar Drugs. 2019 Oct 20;17(10):593. doi: 10.3390/md17100593 (PMC6835275; doi:10.3390/md17100593)
Supplement: Supplementary file 1 [file marinedrugs-17-00593-s001.pdf]

Genome Sequencing of *Streptomyces olivaceus* SCSIO T05 and Activated Production of Lobophorin CR4 *via* Metabolic Engineering and Genome Mining

Chunyan Zhang[1,2], Wenjuan Ding[1,2], Xiangjing Qin[1], Jianhua Ju [1,2,*]

[1] CAS Key Laboratory of Tropical Marine Bio-resources and Ecology, Guangdong Key Laboratory of Marine Materia Medica, RNAM Center for Marine Microbiology, South China Sea Institute of Oceanology, Chinese Academy of Sciences, 164 West Xingang Road, Guangzhou 510301, China; zhchuny@foxmail.com (C.Z.); 13760785354@163.com (W.D.); xj2005qin@126.com (X.Q.)

[2] University of Chinese Academy of Sciences, 19 Yuquan Road, Beijing 110039, China

* Correspondence: jju@scsio.ac.cn (J.J.); Tel./Fax: +86-20-8902-3028 (J.J.)

Supporting Information Table of Contents

Entry	Content	Page/s
1.	**Table S1.** Strains and plasmids used and constructed in this study.	S3
2.	**Table S2.** Primers used in this study.	S4
3.	**Table S3.** Summary of ^{1}H (500 MHz) and ^{13}C NMR (125 MHz) spectroscopic data for compound **1**.	S5
4.	**Figure S1.** Structures of spirotetronate antibiotics.	S6
5.	**Figure S2.** HRESIMS spectrum of **1**.	S7
6.	**Figure S3.** ^{1}H NMR spectrum of **1** in DMSO-d_6.	S7
7.	**Figure S4.** ^{13}C NMR spectrum of **1** in DMSO-d_6.	S8
8.	**Figure S5.** Sequence alignment of LobP2, KijB3 and LbpP2.	S9
9.	**Figure S6.** *LbpC4* disruption in *S. olivaceus* SCSIO T05RX *via* PCR-targeting.	S10
10.	**Supplemental References.**	S11

Table S1. Strains and plasmids used and constructed in this study.

**Strains or plasmids**	**Description**	**Reference or source**
**Strains**		
E. coli LE392	Host strain of cosmid vector SuperCos I	Stratagene
E. coli DH5α	Host strain of general clone	Stratagene
E. coli BW25113	Host strain for PCR-targeting	[1]
E. coli ET12567/pUZ8002	Host strain for conjugation	[2]
S. olivaceus SCSIO T05	Rishirilide-producing strain	This study
S. olivaceus SCSIO T05R	Rishirilides-free strain originated from *S. olivaceus* SCSIO T05	This study
S. olivaceus SCSIO T05RX	Rishirilides/xiamycins-free strain originated from *S. olivaceus* SCSIO T05	This study
S. olivaceus SCSIO T05RXL	Rishirilides/xiamycins/lobophorins-free strain originated from *S. olivaceus* SCSIO T05	This study
**Plasmids**		
SuperCosI	Amp^r, Kan^r, cosmid vector	Stratagene
pIJ790	Cml^r, including λ-RED (*gam*, *bet*, *exo*) for PCR-targeting	[3]
pIJ773	Apr^r, source of acc(3)IV and *oriT* fragment	[3]
pUZ8002	Kan^r, including *tra* for conjugation	[4]
p01-07D	Amp^r, Kan^r, harboring *lbpC4* gene	This study
p21-02E	Amp^r, Kan^r, harboring *lbpC4* gene	This study

Table S2. Primers used in this study.

Primer	Sequence (5`-3`)
**For PCR screening of genomic library**	
lbp-scr-1F	AGCGATCCCAAGGTGACGCT
lbp-scr-1R	ACGGATTCCAGCGAGTCGCA
lbp-scr-2F	ATGGAAACCGAACCCGTCA
lbp-scr-2R	ACCTACCCCTTCCAACACCA
**For construction of gene disruption mutant**	
lbpC4_fw	CTCGCCCCGGTCCCAGTTCAGCGCGTCGAGCATCTCGTCATTCCGG GGATCCGTCGACC
lbpC4_re	GACGTGGACCTCCTCGTCATGGGCACCTCGTCGCCCGACTGTAGG CTGGAGCTGCTTC
lbpC4_Kon_fw	TCCACTTGCTGGACTCGAT
lbpC4_Kon_re	TGAGCGTTTACCTGCACTC
**Sequencing of *lbpP2***	
lbpP2_fw	CGTGGGAACAGCACACGA
lbpP2_re	GACCTGCACGGATTCCAG

Table S3. Summary of ^{1}H (500 MHz) and ^{13}C NMR (125 MHz) spectroscopic data of compound **1** in DMSO-d_6.

position	δ_C type	δ_H mult. (J in Hz)	position	δ_C type	δ_H mult. (J in Hz)
1	167.4, C		**Sugar A**		
2	102.3, C		A-1	97.6, CH	4.72, d (3.4)
3	203.0, C		A-2	32.0, CH_2	1.58, overlapped; 2.29, overlapped
4	50.6, C		A-3	66.9, CH	3.99, overlapped
5	43.0, CH	2.01, overlapped	A-4	70.8, CH	3.22, overlapped
6	30.8, CH	1.58, overlapped	A-5	64.2, CH	3.99, overlapped
7	41.5, CH_2	1.58, overlapped; 1.77, overlapped	A-6	18.0, CH_3	1.18, overlapped
8	34.0, CH	2.29, overlapped	**Sugar B**		
9	83.6, CH	3.37, dd (4.9, 10.0)	B-1	91.3, CH	5.17, overlapped
10	37.9, CH	2.01, overlapped	B-2	35.2, CH_2	1.77, overlapped; 2.01, overlapped
11	125.9, CH	5.82, d (10.4)	B-3	66.3, CH	3.99, overlapped
12	126.3, CH	5.42, m	B-4	82.2, CH	3.22, overlapped
13	52.1, CH	3.53, d (3.8)	B-5	62.0, CH	3.99, overlapped
14	134.3, C		B-6	17.5, CH_3	1.18, overlapped
15	124.0, CH	5.17, overlapped	**Sugar C**		
16	29.3, CH_2	2.29, overlapped	C-1	99.2, CH	4.89, d (9.4)
17	70.9, CH	3.99, overlapped	C-2	37.9, CH_2	1.58, overlapped; 2.01, overlapped
18	140.5, C		C-3	62.0, CH	4.22, d (2.0)
19	117.9, CH	5.17, overlapped	C-4	81.5, CH	2.82, dd (2.2, 9.3)
20	39.9, CH	3.49, d (10.3)	C-5	67.6, CH	3.76, m
21	121.2, CH	5.17, overlapped	C-6	18.3, CH_3	1.18, overlapped
22	136.7, C		C-7	55.7, CH_3	3.22, overlapped
23	31.2, CH	2.29, overlapped			
24	34.5, CH_2	1.77, overlapped; 2.01, overlapped			
25	82.3, C				
26	196.6, C				
27	14.3, CH_3	1.52, s			
28	22.1, CH_3	0.64, s			
29	13.9, CH_3	1.12, d (6.8)			
30	13.7, CH_3	1.34, s			
31	14.6, CH_3	1.37, s			
32	21.6, CH_3	1.80, s			
33	19.6, CH_3	1.24, d (7.1)			

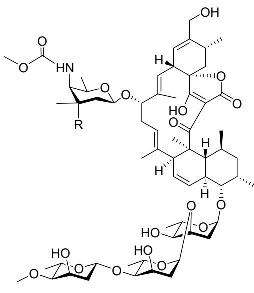

lobophorin A, R = NH_2
lobophorin B, R = NO_2
lobophorin CR1, R = OH

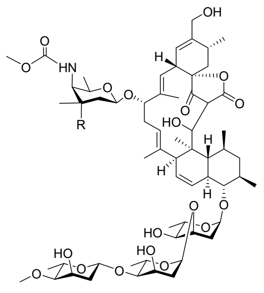

lobophorin C, R = NO_2
lobophorin D, R = NH_2

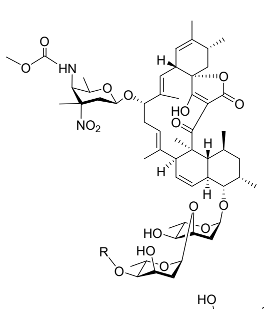

lobophorin E, R =

lobophorin F, R = H

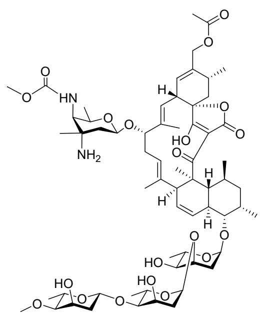

lobophorin G

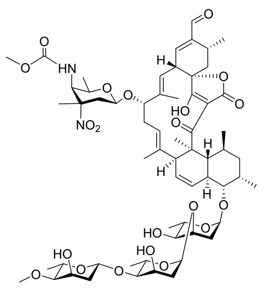

lobophorin H

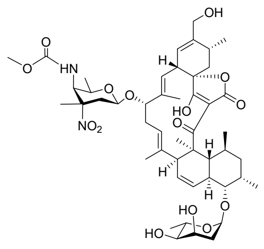

lobophorin I

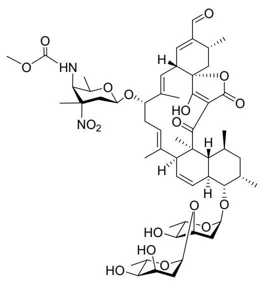

lobophorin J

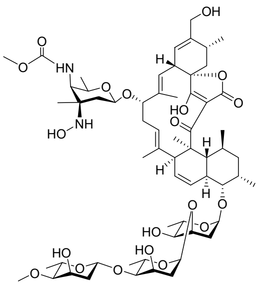

lobophorin K

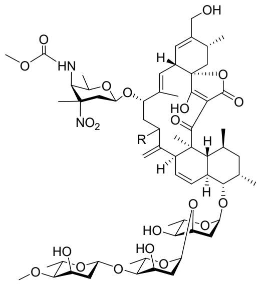

lobophorin CR2, R = OH
lobophorin CR3, R = OOH

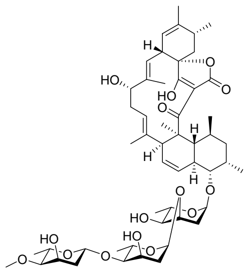

lobophorin CR4 (**1**)

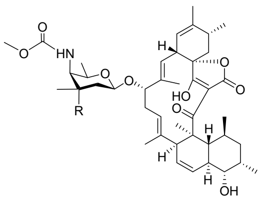

lobophorin CR5, R = NH_2
lobophorin CR6, R = NO_2

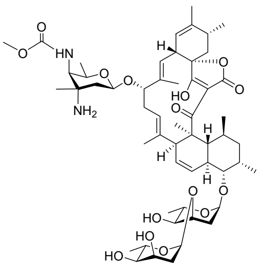

lobophorin CR7

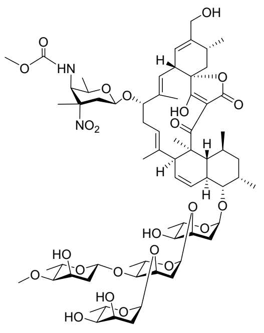

kijanimicin

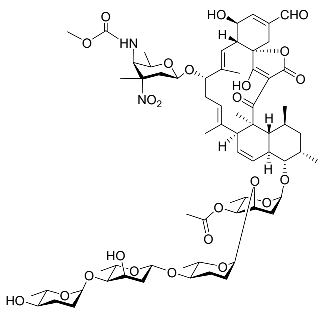

tetrocarcin A

Figure S1. Structures of spirotetronate antibiotics.

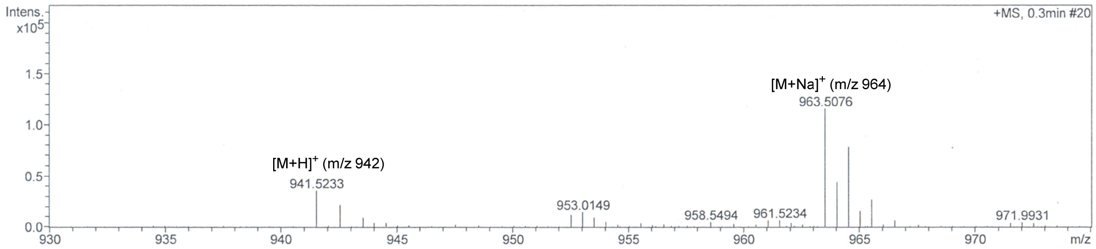

Meas. m/z	#	Formula	Score	m/z	err [ppm]	err [mDa]	mSigma	rdb	e⁻ Conf	N-Rule
941.5233	1	C 52 H 77 O 15	76.82	941.5257	2.5	2.4	44.9	14.5	even	ok
	2	C 65 H 69 N 2 O 4	100.00	941.5252	2.0	1.9	49.7	32.5	even	ok
963.5076	1	C 65 H 68 N 2 Na O 4	100.00	963.5071	-0.5	-0.5	23.2	32.5	even	ok
	2	C 52 H 76 Na O 15	62.95	963.5076	0.1	0.1	47.7	14.5	even	ok

Figure S2. HRESIMS spectrum of **1.**

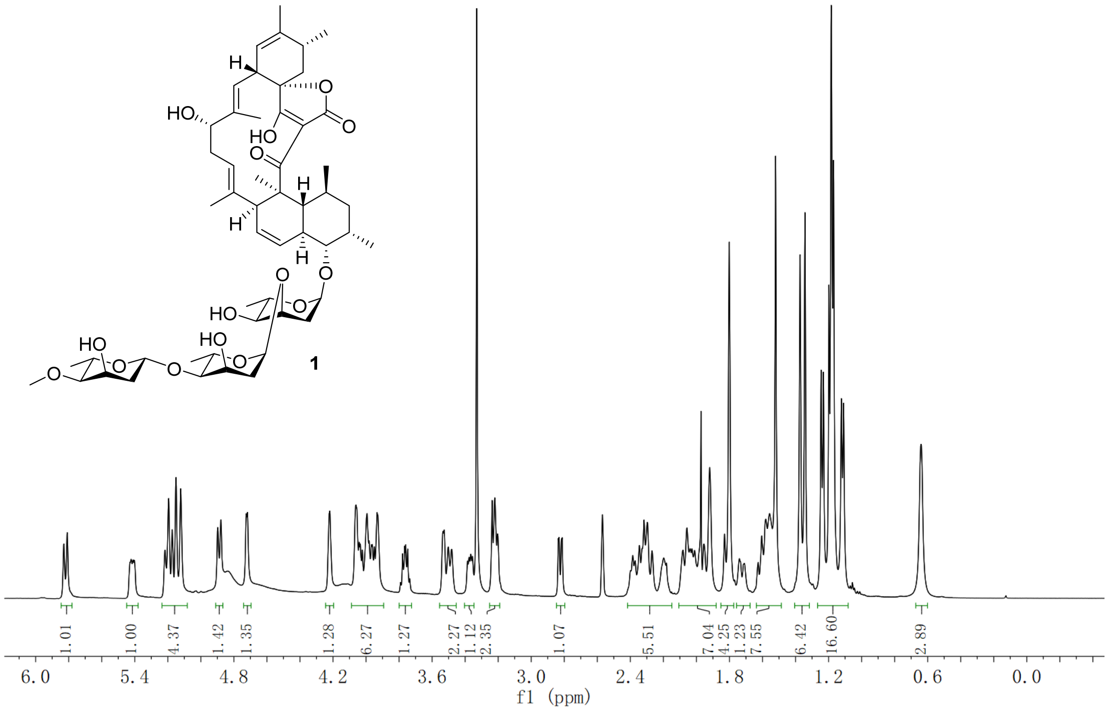

Figure S3. ^{1}H NMR spectrum of **1** in DMSO-d_6.

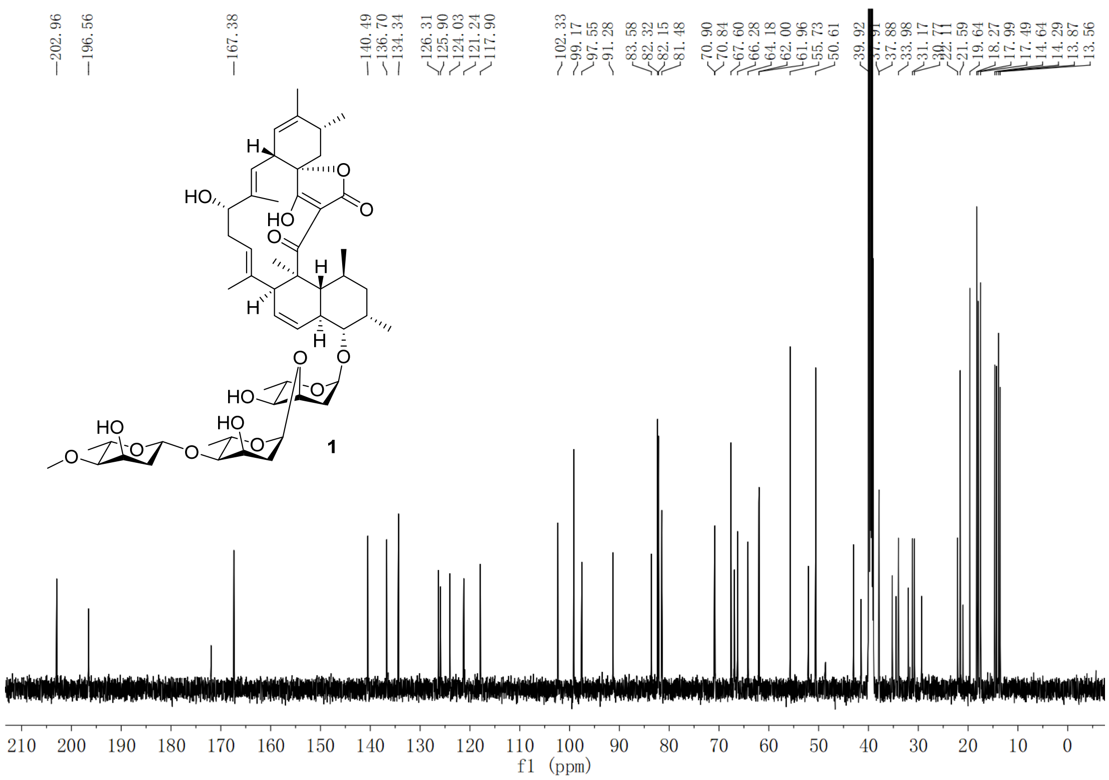

Figure S4. ^{13}C NMR spectrum of **1** in DMSO-d_6.

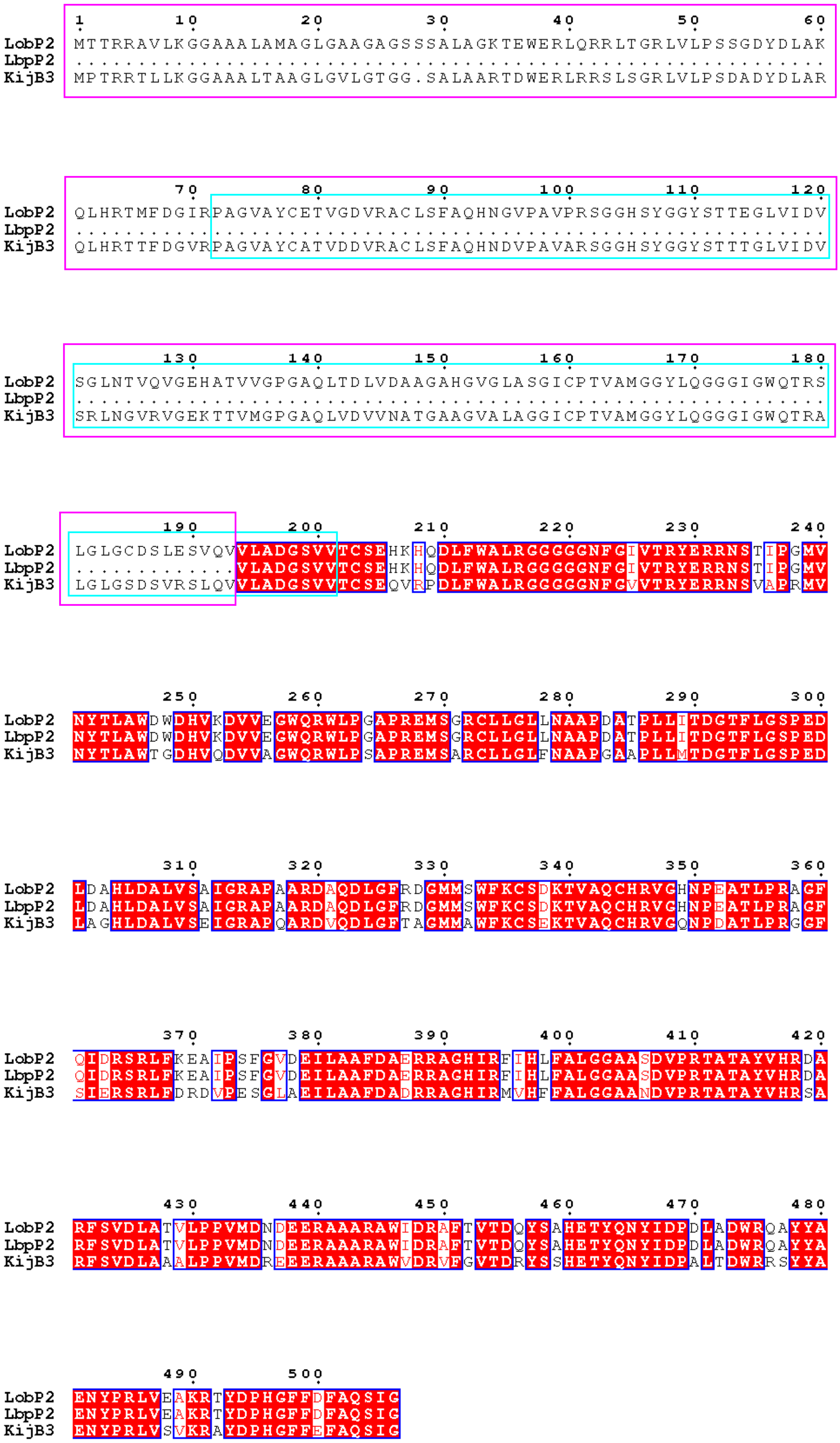

Figure S5. Sequence alignment of LobP2, KijB3 and LbpP2. The FAD binding domains showed in the light blue box; the missing region showed in the pink box.

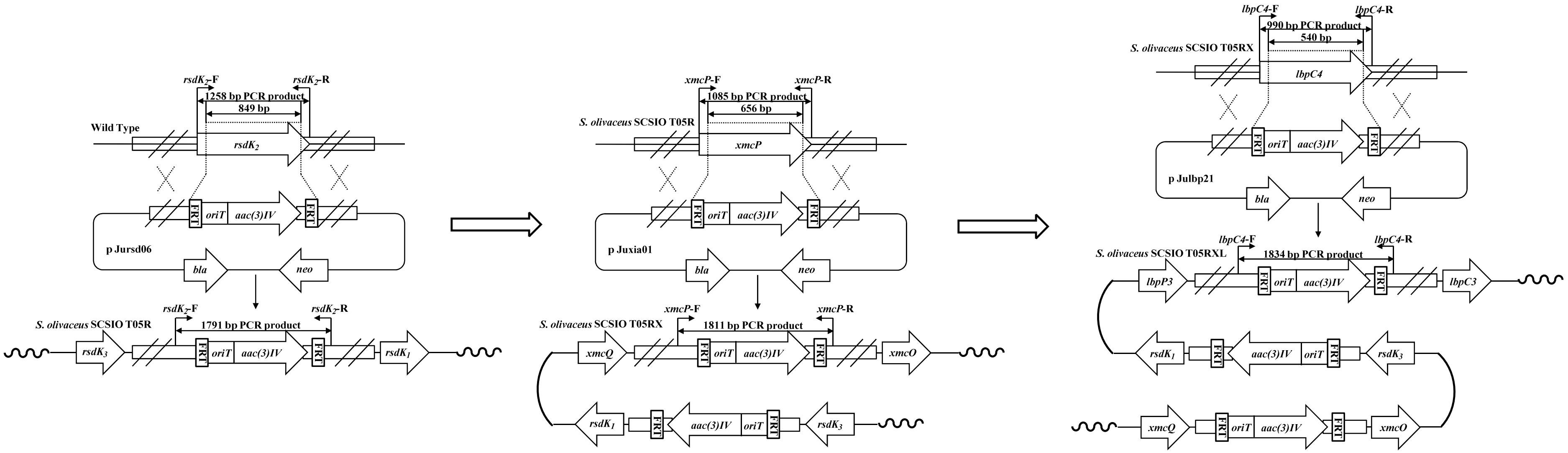

Figure S6. *LbpC4* disruption in *S. olivaceus* SCSIO T05RX *via* PCR-targeting.

Supplemental References.

[1] MacNeil, D. J.; Gewain, K. M.; Ruby, C. L.; Dezeny, G.; Gibbons, P. H.; MacNeil, T. *Gene* **1992**, *111*, 61–68.

[2] Datsenko, K. A.; Wanner, B. L. *Proc. Natl. Acad. Sci. U.S.A.* **2000**, *97*, 6640–6645.

[3] Gust, B.; Chandra, G.; Jakimowicz, D.; Yuqing, T.; Bruton, C. J.; Chater, K. F. *Adv. Appl. Microbiol.* **2004**, *54*, 107–128.

[4] Paget, M. S. B., Chamberlin, L.; Atrih, A.; Foster,S. J.; Buttner, M. J. *J. Bacteriol.* **1999**, *181*, 204–211.
